# Spontaneous Middle Meningeal Artery Aneurysms: A Case Report and Review of the Literature

**DOI:** 10.7759/cureus.49407

**Published:** 2023-11-25

**Authors:** Felipe Ramirez Velandia, Michael Young, Christopher S Ogilvy

**Affiliations:** 1 Neurosurgery, Beth Israel Deaconess Medical Center, Harvard Medical School, Boston, USA; 2 Neurosurgery, Beth Israel Deaconess Medical Center (BIDMC) Brain Aneurysm Institute, Boston, USA

**Keywords:** endovascular coil embolization, spontaneous, middle meningeal artery, endovascular procedures, aneurysm

## Abstract

Most reported aneurysms concerning the middle meningeal artery (MMA) are pseudoaneurysms; however, there have been rare reports of non-traumatic MMA aneurysms in the literature. In this paper, we present the case of a 70-year-old female with a true 5-mm aneurysm in the anterior division of the left MMA that was causing erosion through the left temporal bone and was successfully treated with coil embolization. We also present a comprehensive literature review of non-traumatic MMA aneurysms reported since 1930. These aneurysms are associated with conditions such as hypertension, Paget's disease, and intracranial meningiomas and result from flow dynamics disturbances. Treatment involves both endovascular treatment and open surgery, while rupture, especially in the elderly, results in high disability, underscoring the importance of timely intervention upon identification.

## Introduction

The middle meningeal artery (MMA) is one of the terminal branches of the internal maxillary artery, which continues from the external carotid artery. Its course takes it through the foramen spinosum and along the internal vault of the temporal bone, where it typically bifurcates into two branches: the anterior or frontal, and the posterior or parietal, branch [1]. However, due to its complex embryological development, the MMA exhibits variations and anomalies in terms of origin, branches, and positional characteristics, as documented in the literature [2].

The MMA holds significant importance in the field of endovascular neurosurgery, as it is commonly used for treating conditions such as dural arteriovenous fistulas (DAVF), meningiomas, and more recently, chronic subdural hematomas (CDSH) [3,4]. While the majority of reported aneurysms concerning MMA are pseudoaneurysms resulting from trauma, endovascular procedures, or surgical interventions [5,6], a primary MMA aneurysm has been reported rarely in the literature [4]. In this paper, we present a case of a non-traumatic MMA aneurysm and review the reported cases of these aneurysms in the literature.

## Case presentation

**Figure 1:**
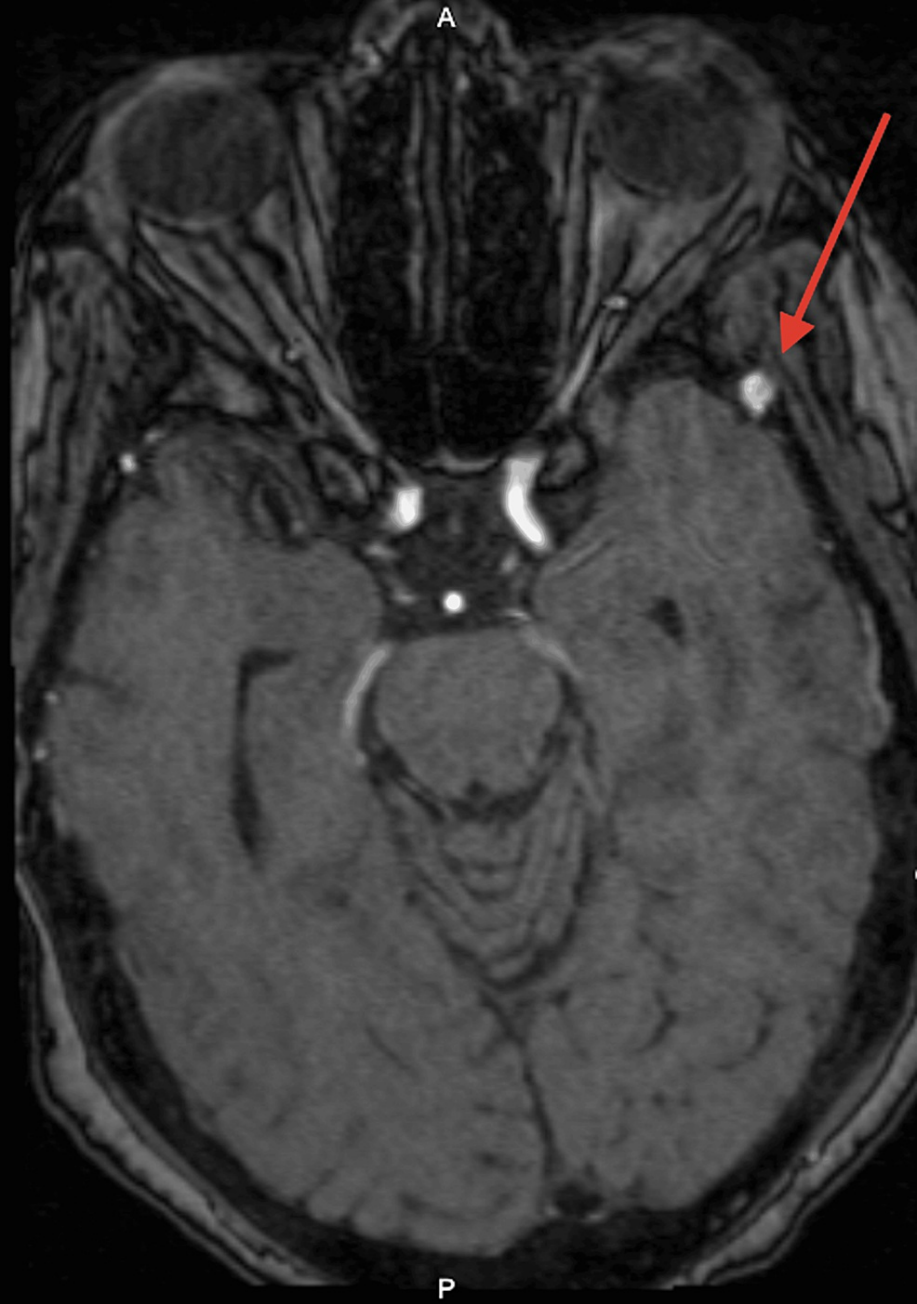
Axial magnetic resonance angiography (MRA) Demonstrates an aneurysm of the left middle meningeal artery.

A 70-year-old female with a history of hypertension and asthma presented with long-standing left-sided pulsatile headaches over the pterion. There was no clear history of trauma. Upon examination, she was neurologically intact, without any cranial nerve, motor, or sensory deficits. Magnetic resonance angiography identified an approximately 5 mm left anterior division MMA aneurysm (Figure 1), situated in the region associated with her left temporal headaches. The case was discussed in the weekly multidisciplinary neurovascular conference, and the decision was made to pursue endovascular treatment due to the aneurysm's location, the potential risk of epidural hematoma, and the patient's overall good functional status.

**Figure 2:**
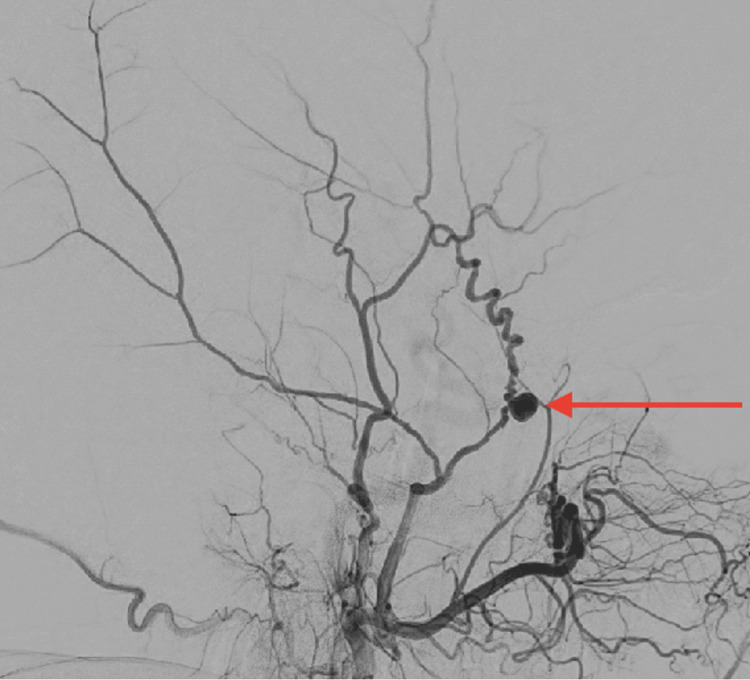
Lateral digital subtraction angiography (DSA) DSA displaying a saccular aneurysm of the anterior division of the left middle meningeal artery.

**Figure 3:**
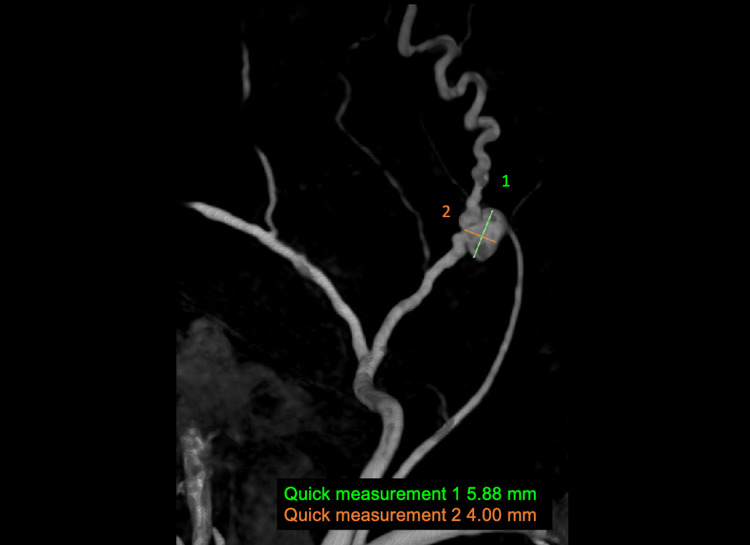
Three-dimensional angiography 5.88 mm × 4 mm saccular aneurysm of the anterior division of the left middle meningeal artery can be visualized.

On the day of the elective procedure, the patient was transferred to the neuro-angiography table, and she received a pre-procedure dose of prednisone as she reported an allergy to iodine contrast. Initial diagnostic cerebral angiography, including 3D acquisitions, was performed, revealing a 5.88 mm × 4.0 mm aneurysm arising from the left anterior (frontal) division of the MMA (Figures 2-3). Notably, distal to the aneurysm, the MMA exhibited a highly tortuous course. The aneurysm was subsequently embolized with three Stryker target 360 soft 4 mm × 15 cm detachable coils.

**Figure 4:**
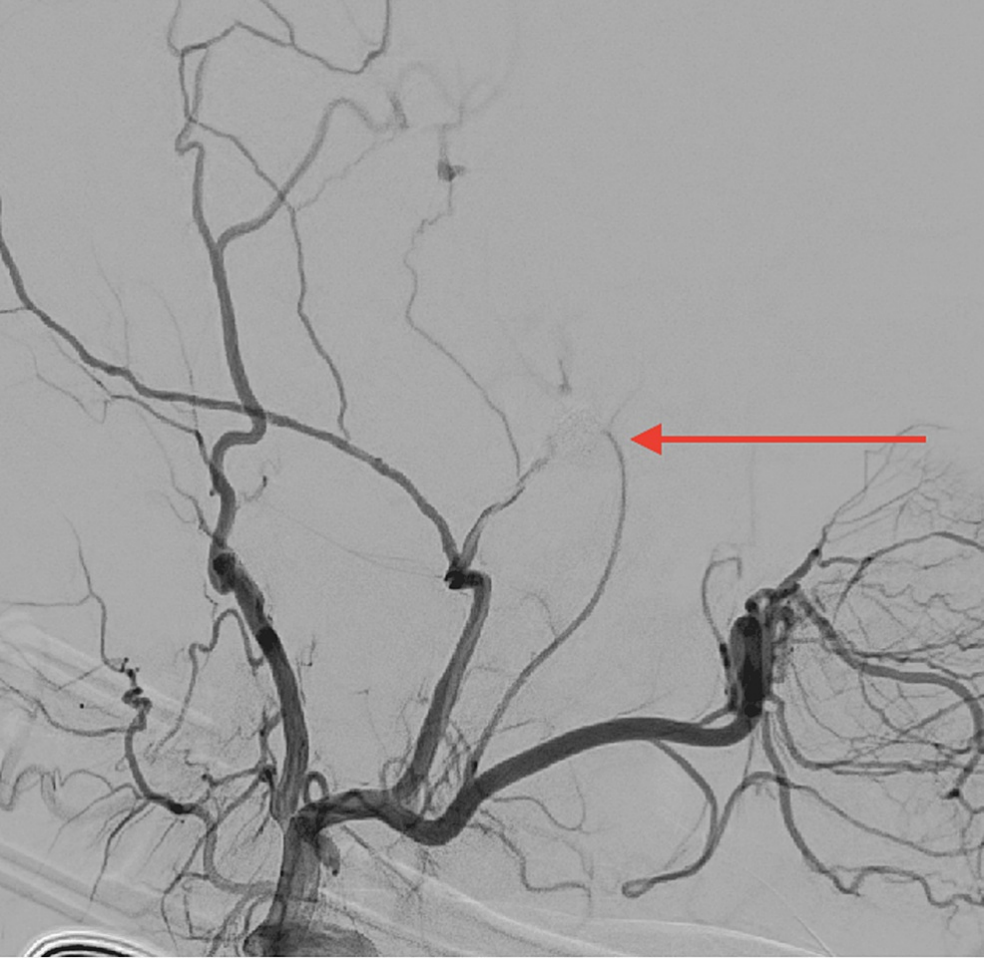
Post-coil embolization lateral digital subtraction angiography (DSA) Showing complete obliteration of the anterior branch left middle meningeal artery aneurysm.

Post-coiling left external carotid artery angiogram revealed a complete occlusion (Raymond Roy 1) of the aneurysm, with no distal filling of the MMA (Figure 4). Subsequent assessment of the ipsilateral internal carotid artery and the posterior circulation revealed no major vascular anomalies or intraprocedural thromboembolic complications. Subsequently, the patient was transferred to the post-anesthesia care unit (PACU) and then to the neurocritical care unit.

The following day, the patient's neurological exam remained intact, with normal labs and vitals. She was tolerating a regular diet, mobilizing independently, and voiding without difficulty. Her pain was well controlled, and she was medically cleared for discharge home. Two weeks after the procedure, she was seen in the neurosurgery clinic, reporting a complete resolution of her headaches. She will be evaluated with additional imaging after completing one to two years of follow-up to assess complete occlusion and rule out recurrence.

## Discussion

Methods

**Figure 5:**
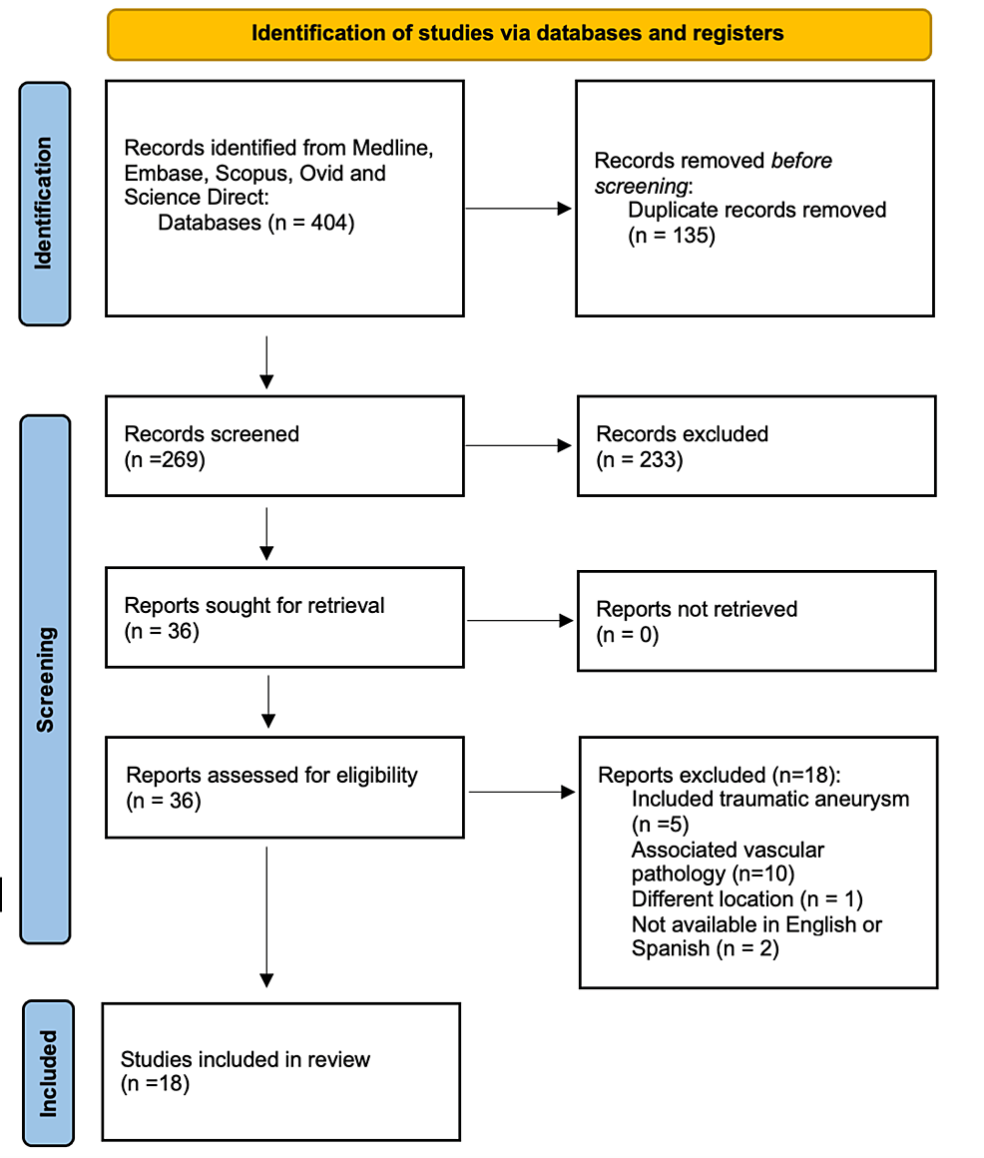
Preferred Reporting Items for Systematic Reviews and Meta-Analyses (PRISMA) flow chart Detailed flow chart of the selection process of the articles included in the literature review

We conducted a comprehensive search of multiple databases, including PubMed, Embase, Scopus, Ovid, and ScienceDirect, to conduct a thorough literature review of middle meningeal artery aneurysms. Our search encompassed articles published from January 1930 to September 2023. The following search strategy was employed: ‘‘middle meningeal artery” AND “aneurysm.” We screened articles that met the following criteria: (1) patients with non-traumatic middle meningeal artery aneurysms diagnosed angiographically; (2) articles written in English or Spanish; and (3) availability of the full text for review. The search identified 269 articles after removing duplicates, of which 36 met the inclusion criteria. After a thorough reading of the selected articles, 18 manuscripts were excluded: two were written in a different language, five reported traumatic aneurysms, 10 reported associated vascular lesions (arteriovenous malformation, dural arterio-venous fistula, cavernomas, arterial occlusion, and Moya moya disease), and one reported an aneurysm at the junction between the internal carotid artery and the posterior communicating artery (refer to Figure 5).

Results

We found a total of 18 cases of non-traumatic MMA aneurysms reported in the literature since 1930 (Table 1). Most of these cases involved females in their fourth decade of life or older. A significant number of the reported cases presented with loss of consciousness secondary to aneurysm rupture [7-11], with a variable presentation at the site of the bleeding (Table 2). Aneurysms were predominantly located in the proximal MMA [9,12-16], and the size was less than 10 mm for most cases, with the exception of a giant MMA aneurysm that was reported in a patient with fibrous dysplasia [14]. Some of the conditions associated with the reported cases of spontaneous MMA aneurysm were hypertension [9,12,13,17,18], neurofibromatosis type 2 [19], Paget’s disease of the bone [12,17,18], and intracranial meningiomas [15,19-23]. In terms of management, most unruptured cases underwent endovascular treatment with glue embolization [10,14,19]. For cases reporting an associated intracranial hemorrhage, a craniotomy with ligation and coagulation was performed [8,9,11-13,24]. Regarding outcomes, most patients recovered to baseline when identified and treated before rupture. However, half of those presenting with hemorrhagic stroke [9,11,24] experienced some long-term disability.

**Table 1:** Literature review the reported cases of non-traumatic MMA aneurysms MMA: middle meningeal artery; CAD: coronary artery disease; HTN: hypertension; DM: diabetes mellitus; Afib: atrial fibrillation; UMN: upper motor neuron; PCA: posterior cerebral artery; SAH: subarachnoid hemorrhage; SDH: subdural hemorrhage; EDH: epidural hematoma; ICH: intracranial hemorrhage; IVH: intraventricular hemorrhage; NF-1: Neurofibromatosis type 2; L: left; R: right; F: females; M: males.

Reference	Age/sex	Presentation	History	Aneurysm status	Aneurysm location	Additional features	Hemorrhage	Associated intracranial lesions at admission	Treatment	Outcome
Harvie et al. [7]	21/M	Loss of consciousness	None	Ruptured	N/A	Ruptured fusiform	L EDH	None	N/A	Died
Berk et al. [12]	73/F	Headaches, pulsatile mass	HTN, Paget disease	Unruptured	L proximal MMA	Saccular, bony erosion	None	None	Craniotomy, ligation + resection	Recovery to baseline
Holland and Thomson [8]	49/F	Headaches, loss of consciousness	None	Ruptured	R posterior MMA	R MMA, posterior branch	R EDH, SAH	None	Craniotomy, ligation + resection	Recovery to baseline
New [17]	79/F	Loss of consciousness	DM, HTN, CAD, Afib, PAD, Paget disease	Unruptured	R distal MMA	Saccular (pear-shaped), 5 mm × 7 mm	None	None	N/A	N/A
New [18]	57/F	Bifrontal headaches, pulsatile mass	HTN, Paget disease, Syphilis	Unruptured	R anterior MMA	Saccular, partially thrombosed	None	None	Medical management, patient refused surgical ligation	No change
Bollati et al. [16]	50/F	Seizure	None	Unruptured	L proximal MMA	N/A	None	None	Craniotomy, ligation + resection	Recovery to baseline
Korosue et al. [24]	66/F	Sudden onset severe headache, recurred after surgery	None	Ruptured	L posterior MMA	Saccular	SDH	None	Craniotomy, ligation + resection	Improvement of symptoms
O'Neill et al. [20]	82/F	Left UMN syndrome	None	Unruptured	R bifurcation	Saccular	None	Convexity meningioma	Coiling	Improvement of symptoms
Muras et al. [21]	57/M	Headache and progressive left-sided weakness.	None	Unruptured	R distal MMA	Saccular, small	None	Convexity meningioma	Surgical resection of meningioma	Recovery to baseline
Sandin et al. [9]	46/M	Sudden onset of headache and loss of consciousness.	HTN	Ruptured	R proximal MMA	Saccular, 8 mm	R temporal ICH	None	Craniotomy, ligation + resection	Improvement of symptoms
Lama and Mottolese [22]	69/F	Mental confusion	None	Unruptured	R bifurcation	N/A	None	Pterional meningioma	Coiling and PVA for feeding vessels of the tumor	Recovery to baseline
Kobata et al. [11]	77/F	Sudden loss of consciousness + right hemiparesis	None	Ruptured	R distal anterior MMA	Two saccular aneurysms (0.3 and 0.8 mm)	L frontal ICH	None	Craniotomy, ligation + resection	Improvement of symptoms
Lesley et al. [19]	36/M	Weakness of his right upper extremity	NF-2	Unruptured	R anterior MMA	Saccular, 4-mm	None	Multiple meningiomas (parafalcine, bilateral sphenoid wings, and tentorium)	PVA embolization	Improvement of symptoms
Maekawa et al. [23]	72/F	Dizziness	None	Unruptured	L bifurcation	Saccular	None	Convexity meningioma	NBCA glue + lipidol	Improvement of symptoms
Hedjoudje et al. [10]	57/F	Sudden onset vertigo and loss of consciousness	Small cell lung cancer, CAD	Ruptured	R anterior MMA	Saccular	R temporal EDH + SAH	None	NBCA glue	Recovery to baseline
Kpelao et al. [13]	57/M	Drug-resistant epilepsy	HTN	Unruptured	R proximal MMA	Saccular, wide neck	None	None	Craniotomy, ligation + resection	Recovery to baseline
Yamashita et al. [14]	66/F	Reduced visual acuity	Fibrous dysplasia	Unruptured	L proximal MMA	Giant thrombosed, multiple small distal	None	Osteolytic parietal lesion	NBCA glue	Recovery to baseline
Carlstrom et al. [15]	75/F	Headaches	None	Unruptured	L proximal MMA	Saccular, 6.1 mm × 6.7 mm × 4.8 mm	None	L frontal giant meningioma (7.5 cm × 6.7 cm × 5.9 cm)	PVA embolization	Recovery to baseline
Our case	70/F	Temporal headaches	HTN, asthma	Unruptured	L anterior MMA	Saccular, bony erosion, 5.88 mm × 4.0 mm	None	None	Coil embolization	Recovery to baseline

**Table 2:** Summary of key findings from reported cases of non-traumatic MMA aneurysms in the literature *Commonly associated conditions. IQR: interquartile range; NF-2: neurofibromatosis type 2; ICH: intracranial hemorrhage; EDH: epidural hematoma; SAH: subarachnoid hemorrhage; SDH: subdural hematoma; PVA: polyvinyl alcohol.

Non-traumatic MMA aneurysms (N=18)	
Age	
Median (IQR)	61.5 (50-73)
Sex	N (%)
Females	13 (72.22)
Males	5 (27.78)
Presentation	N (%)
Headaches	6 (33.33)
Loss of consciousness	5 (27.78)
Seizure	2 (11.11)
Motor deficit	2 (11.11)
Confusion	1 (5.56)
Visual disturbances	1 (5.56)
Vertigo	1 (5.56)
History	N (%)
Hypertension*	5 (27.78)
Paget disease*	3 (16.67)
NF-2	1 (5.56)
Syphilis	1 (5.56)
Fibrous dysplasia	1 (5.56)
Associated lesion	N (%)
Meningioma*	6 (33.33)
Status	N (%)
Unruptured	12 (66.68)
Ruptured	6 (33.33)
ICH	2 (11.11)
EDH	2 (11.11)
SAH	2 (11.11)
SDH	1 (5.56)
Location	N (%)
Proximal	6 (33.33)
Anterior	3 (16.67)
Posterior	2 (11.11)
Bifurcation	3 (16.67)
Distal	3 (16.67)
Management	N (%)
Craniotomy	7 (38.89)
Endovascular	7 (38.89)
Coils	2 (11.11)
Glue	3 (16.67)
PVA	2 (11.11)

Discussion

The first case of a primary MMA aneurysm was reported by Harvie in 1930 when he described a spontaneous epidural hematoma arising following a rupture of a fusiform MMA aneurysm; unfortunately, this patient died [7]. Three decades later, Berk pointed out three findings that he considered essential for the diagnosis of a congenital MMA aneurysm: a cortical bony defect, a residual superficial temporal artery, and a dilated MMA [12].

Following Berk's observation, in 1967, New P.F. proposed a triad of symptoms for spontaneous aneurysms in the MMA. This triad included hypertension, Paget's disease of the bone, and a proximal intracranial aneurysm in the middle meningeal arterial tree [18]. New P.F. hypothesized flow dynamics disturbances in the MMA secondary to the cortical thickening seen in Paget disease. New pioneered the concept that an MMA aneurysm arises in a state of increased overall resistance and wall stress within the inner vault of the temporal bone.

Increasingly, reports of primary spontaneous MMA aneurysms in patients with meningiomas are available in the literature [15,20,22,23]. Carlstrom et al. recently described a patient with a giant meningioma who was incidentally found to have an MMA aneurysm [15]. This suggests that the development of MMA aneurysms could be linked to increased flow demands in feeding arteries and the neo-angiogenesis associated with vascularized lesions like meningiomas, aligning with the flow disturbance principle introduced by New. Beyond meningiomas, MMA aneurysms have been reported in association with vascular anomalies, including dural arterial venous malformations, angiomas, and Moya Moya disease [19,25-29].

The hypothesized multifactorial etiology of MMA aneurysms involves an interplay of factors such as reduced support of the MMA, increased blood flow, and chronic systemic hypertension [17]. This is thought to occur over an extended period, explaining the rarity of this condition. However, exceptions to this pattern may arise. For instance, Jin et al. reported the case of a nine-year-old child who presented with left-sided headaches and was found to have an unruptured pear-shaped aneurysm and angioma in the distal part of the anterior MMA [30]. This case suggests that genetics may play a role in the development of an MMA aneurysm as well as general intracranial aneurysms [31].

While the mortality rate associated with ruptured MMA aneurysms appears to be lower compared to other intracranial aneurysms [32], the morbidity remains significant, with long-term disability reported in a substantial portion of cases [9,11,24]. Consequently, the current trend leans towards prompt intervention upon the identification of MMA aneurysms. Endovascular management emerges as an alternate approach, with positive outcomes reported for various embolic agents such as glue, coils, and PVA [10,14,15,19,20,22,23]. However, positive results were also seen in patients with unruptured aneurysms undergoing craniotomy [13,16], which can be minimally invasive with a small burr hole. In our case, we report the use of primary coiling with angiographic obliteration and symptom resolution. The key takeaway remains the importance of timely treatment upon identification.

## Conclusions

Primary middle meningeal artery aneurysms are exceedingly rare. The reported aneurysms have predominantly been located in the proximal MMA and have been associated with hypertension, Paget's disease, and intracranial meningiomas. MMA aneurysms result from an interplay between flow disturbances and shearing stress during the course through the inner vault of the temporal bone. The primary approach includes both endovascular embolization and open resection, with the latter being possible through a burr hole. Prompt intervention prior to rupture is essential, especially in the elderly, where rupture leads to high disability. This highlights the importance of early recognition and intervention for improved clinical outcomes.
